# 

**Published:** 1880-01

**Authors:** 


### CONTENTS FOR JANUARY, 1880.

ORIGINAL COMMUNICATIONS.

ART. I.—Compression and Immobilization Principal Factors in Surgery. By GEORGE HALSTED BOYLAND, A. M., M. D. .......... 1

ART. II.—Water—Its Relation to Health and Disease. By WOOTEN M. WILKERSON, A. B., M. D .......... 4

ART. III.—A "Speedy Method" in Asphyxia. By HARVEY L. BYRD, M. D. .......... 11

ART. IV.—Clinical Notes upon the use of the Galvano-Cautery. By WM. A. BYRD, M. D .......... 14

ART. V.—Tetanus—Epidemic or Constitutional among Negroes? By R. H. GOLDSMITH, M. D .......... 22

ART. VI.—Our Young Dental Patients. By CHARLES E. FRANCIS, D. D. S. .......... 24

ART. VII.—Teeth, Pregnancy and Disease. By BASIL M. WILKERSON, D. D. S., M. D .......... 26

SELECTIONS.

Extirpation of the Bones of the Nose and Mouth by the use of the Surgical Engine. By D. H. GOODWILLIE, M. D., D. D. S. .......... 28

On the Therapeutics of Acute Rheumatism. By ROBERTS BARTHOLOW, M. D. 37

An Intractable Molar. By P. K. STODDARD, M. D .......... 44

A Pleasant Remedy for Toothache. By T. C. OSBORN, M. D .......... 49

Obtunding Sensitive Dentine .......... 52

EDITORIAL.

Prospectus .......... 45

Life Insurance Associations .......... 46

Advertisers, Communications, etc .......... 47

Translations, Books, Pamphlets, etc .......... 48

Mortuary Statistics .......... 50

Report of Signal Service at Baltimore .......... 52

